# The D826V point mutation in *IREB2* induces lipogenesis in adipose tissues

**DOI:** 10.3389/fcell.2026.1724485

**Published:** 2026-01-30

**Authors:** Yibing Lv, Chunyu Wu, Junyi Xiao, Wenke Yang, Chenyang Wang, Jinming Wang, Jianmei Huang, Zhenglong Guo, Shixiu Liao

**Affiliations:** 1 Henan Provincial Key Laboratory of Genetic Diseases and Functional Genomics and Medical Genetics Institute of Henan Provincial People’s Hospital, Henan Provincial People’s Hospital, People’s Hospital of Zhengzhou University, Zhengzhou, China; 2 School of Medicine, People’s Hospital of Zhengzhou University, Zhengzhou University, Zhengzhou, China; 3 State Key Laboratory of Natural Medicines, School of Traditional Chinese Pharmacy, China Pharmaceutical University, Nanjing, China; 4 School of Basic Medicine, Tongji Medical College, Huazhong University of Science and Technology, Wuhan, Hubei, China

**Keywords:** fatty acid, ferritin, IREB2, lipid biosynthesis, triglyceride

## Abstract

**Background:**

IREB2 is an RNA-binding protein that modulates cellular iron uptake and storage by regulating ferritin expression. Mutations in the *IREB2* gene have been shown to induce degradation of the IREB2 protein, resulting in the upregulation of FTH1 protein expression, which contributes to iron storage. This study investigates the potential mechanism of the *Ireb2* ^D826V/D826V^ mutation on lipid biosynthesis for the first time.

**Results:**

In this study, it was discovered for the first time that *Ireb2* ^D826V/D826V^ mice exhibited increased body weight, hypertrophic adipocytes, iron and lipid accumulation. RNA-sequencing analysis suggests that the *Ireb2* ^D826V/D826V^ mutation potentially activates pathways involved in fatty acid and triglyceride biosynthesis. This activation is evidenced by the upregulation of lipid biosynthetic genes, including *Fasn*, *Acaca*, *Acly*, *Fgfr4*, and *Egr1*, along with their corresponding protein expressions in *Ireb2* ^D826V/D826V^ mice. These molecular changes occur alongside the degradation of IREB2, an increase in FTH1 expression and iron accumulation in tissues. Metabolomic analysis confirmed that the *Ireb2* ^D826V/D826V^ mutation indeed elevated the levels of certain fatty acids and triglyceride components in epWAT. Moreover, *in vitro* experiments utilizing adipocytes confirmed that the *Ireb2* ^D826V/D826V^ mutation augmented fatty acid and triglyceride biosynthesis, as well as the expression of associated proteins, contingent upon iron accumulation.

**Conclusion:**

The *Ireb2* ^D826V/D826V^ mutation is linked to the degradation of IREB2, increased expression of FTH1, and iron accumulation. These molecular alterations are associated with elevated protein levels of ACACA, ACLY, FASN, EGR1, and FGFR4, which correlate with enhanced lipid biosynthesis and subsequent weight gain. This study elucidates a link between dysregulated iron storage, mediated via the IREB2-FTH1 axis, and the upregulation of key lipogenic genes, resulting in enhanced lipid biosynthesis. These findings underscore a potential relationship between iron metabolism and obesity, suggesting that iron homeostasis could serve as a valuable target for further mechanistic investigation and therapeutic development in the context of obesity.

## 1 Introduction

In recent years, alterations in dietary patterns, environmental factors, and genetic predisposition have emerged as significant contributors to the rising prevalence of obesity (Bluher, 2019). Among these factors, genetic susceptibility is the most prominent and uncontrollable determinant (Goodarzi, 2018). Given the relationship between genetic factors and obesity is partially understood, there remains an urgent need to elucidate the underlying mechanisms by which genetic factors contribute to the development of obesity (Goodarzi, 2018).

IREB2 (iron responsive element binding protein 2), an RNA-binding protein that regulates cellular iron uptake and storage, plays a crucial role in coordinating iron homeostasis by interacting with iron-responsive elements (IREs) in target mRNAs, including those encoding the transferrin receptor (TFRC) and the heavy and light chains of ferritin (FTH1 and FTL) (Yanatori et al., 2021; Yao et al., 2024). Mutations in the *IREB2* gene have been shown to induce degradation of the IREB2 protein, subsequently leading to an upregulation of FTH1 expression, which may contribute to the iron accumulation in cells and tissues (Yao et al., 2024; Guo et al., 2024).

A growing body of evidence indicates that iron accumulation in tissues may contribute to the pathogenesis of metabolic diseases such as obesity, type 2 diabetes, hyperlipidemia, and non-alcoholic fatty liver disease. These conditions are clearly associated with exceed lipid biosynthesis in metabolic tissues (Hilton et al., 2023). However, there is a paucity of studies exploring the potential mechanisms linking iron accumulation to lipid biosynthesis. Previous studies proposed that iron overload may increase the FTH1 expression and activate the sgk-1/FATP1/4 signaling pathway, which enhanced the processes of fatty acid uptake and translocation. However, the specific mechanisms through which excess iron and FTH1 activates these signaling pathways and subsequently influences lipid biosynthesis remain unclear. The observed increase in fatty acid uptake and translocation, when considered independently, does not fully elucidate the implications for lipid accumulation within the tissues associated with the aforementioned pathological condition (Wang et al., 2016).

FTH1 directly enhances the accumulation of Fe^3+^ iron while reducing the levels of free Fe^2+^ iron under conditions of iron overload. The elevated expression of FTH1 has been positively correlated with the development of obesity and the process of adipogenesis (Wang et al., 2016). Individuals with obesity and higher waist circumference frequently exhibit higher FTH1 expression (Hilton et al., 2023). The evidence suggests that FTH1 may play a direct role in modulating lipid biosynthesis by regulating iron accumulation; It is reasonable to postulate that the elevated expression of FTH1, which induces iron accumulation, may directly enhance the lipid biosynthetic process, consequently promoting lipid accumulation. However, the precise mechanism by which FTH1 influences lipid biosynthesis remains unclear. To elucidate the specific mechanisms underlying the role of FTH1 in lipid biosynthesis, it has been identified that FTH1 regulated processes of lipogenesis and differentiation in 3T3-L1 adipocytes (Yang et al., 2025; Festa et al., 2000). Furthermore, FTH1 deficiency appears to mitigate disorders associated with high-fat-induced lipid accumulation and obesity (Ikeda et al., 2020). This effect, however, is achieved by inhibiting the inflammatory activation of macrophages when FTH1 is specifically knocked out in these cells, leaving direct evidence of FTH1’s role in lipid biosynthesis still lacking. Based on the aforementioned evidence, it is reasonable to hypothesize that the degradation of IREB2, which induces the upregulation of FTH1 expression, may significantly contribute to the direct stimulation of lipid biosynthesis. This study aims to investigate the underlying mechanisms involved.

In our prior research, we identified a novel *IREB2* c.2477A>T (p.D826V) variant in a patient diagnosed with NDCAMA (Neurodegeneration, Early-onset, with Choreoathetoid Movements and Microcytic Anemia) and exhibiting an abnormal body weight. This variant is predicted and observed to destabilize IREB2 protein by promoting its degradation. To investigate its pathogenic role, we generated *Ireb2* ^D826V/D826V^ mice using CRISPR-Cas9 technology. Notably, during the feeding trials, *Ireb2* ^D826V/D826V^ mice demonstrated increased body weight, enhanced fat mass, and hypertrophic adipocytes compared to wild type (WT) mice of the same age. We hypothesize that the *Ireb2* ^D826V/D826V^ mutation may influence lipid metabolism of adipocytes, but the potential mechanism remains obscure.

In this study, we integrated transcriptomic sequencing with untargeted metabolomics to examine the influence of the *Ireb2* ^D826V/D826V^ mutation on lipogenic and lipolytic processes. Furthermore, both *in vivo* and *in vitro* experiments were performed to elucidate the associated lipid metabolic processes and underlying mechanisms. Our findings offered mechanistic insights into the pathophysiology of obesity and the role of *Ireb2* ^D826V/D826V^ mutation.

## 2 Materials and methods

### 2.1 Animal

The *Ireb2* ^D826V/D826V^ knock-in mouse model was developed using the CRISPR-Cas9 genome editing system by Cyagen Company (Guangzhou, China), employing a strategy consistent with our prior studies (Guo et al., 2025). Initially, the BAC clone RP23-382D20 served as the template for creating the homologous recombination donor DNA, which included the mouse *Ireb2* genomic sequence with the D826V mutation, amplified using high-fidelity Taq DNA polymerase. Two sgRNAs were designed to target the mouse *Ireb2* gene: sgRNA1: 5′-CTT​AGG​TAT​ATA​GTC​AAG​GCT​GG-3′ and sgRNA2: 5′-AAT​ACA​GAT​GAC​ACG​AGA​CAG​GG-3’. A mixture comprising these single-guide RNAs (sgRNAs), each at a concentration of 2 pmol/μL, the homologous recombination template DNA at 15 ng/μL, and the Cas9 protein at 30 ng/μL (M0646M, New England Biolabs, Ipswich, United States), was prepared and subsequently microinjected into fertilized mouse zygotes to generate F0 generation heterozygous mutants. The F0 heterozygous mice were intercrossed to produce F1 offspring. Homozygous *Ireb2* ^D826V/D826V^ mice were identified through genomic DNA extraction from tail tissue, followed by polymerase chain reaction (PCR) amplification and Sanger sequencing. The primers used for sequencing were: Forward: 5′-CAA​GAG​GCA​CAT​TTG​CAA​ACA​TCA-3′ and Reverse: 5′-CTT​CAC​TGC​GTC​CAT​ACA​GAC​TTC-3’. Both the *Ireb2* ^D826V/D826V^ mice (5 mice each group) and their age-matched WT counterparts (5 mice each group) were maintained in specific pathogen-free (SPF) conditions. The animals were housed in cages under a 12 h light/dark cycle within a temperature-controlled environment set at 22 °C ± 2 °C, with a relative humidity of 55% ± 15%. Each cage accommodated four mice and was equipped with a cage frame, a water bottle, and a supply of standard diet (MD17121, Medicience, China, Crude Protein: 25.0%, Crude Fat:10.0%, Crude Fiber:5.0%, Calcium:1.5%, Phosphorus:1.0%). All mice were unrestricted access to standard food and water. In this study, the body weight of WT and *Ireb2* ^D826V/D826V^ mice was monitored weekly starting at 8 weeks of age. Body weight measurements were systematically recorded at 8 weeks (onset of young adulthood), 12 weeks (young adulthood), and 16 weeks (mid-adulthood) to elucidate differences between WT and *Ireb2* ^D826V/D826V^ mice. These time points were strategically chosen to capture the metabolic profile during early adulthood and to monitor the progression of any potential phenotypic changes over time. At 16 weeks of age, representing full adulthood, all mice underwent intraperitoneal glucose tolerance tests (IPGTT) and insulin tolerance tests (ITT). Mice undergo a fasting period, typically lasting 12 h for the IPGTT and 4 h for the ITT. Baseline blood glucose levels are determined from the tail vein at time zero. For the IPGTT, a glucose solution (G8150, Solarbio, China) at a dosage of 2 g/kg body weight was administered intraperitoneally. Subsequent blood glucose measurements are taken at 30, 60, 90, and 120 min post-injection to monitor glucose clearance. In the ITT, insulin (I8040, Solarbio, China) was administered at a dosage of 0.75 U/kg body weight. Blood glucose levels are then measured at 15, 30, 60, and 90 min to evaluate the hypoglycemic response.

Subsequently, all mice were under isoflurane anesthesia for euthanasia, and epididymal adipose tissue (epWAT) and subcutaneous adipose tissue (SAT) were harvested for further analyses, including the calculation of fat mass (ratio of adipose tissue weight to body weight), hematoxylin and eosin (H&E) staining, western blot analysis, metabolomic profiling, and RNA sequencing. Additionally, serum and epWAT samples were collected to measure triglyceride (TG) and total cholesterol (TC) concentrations.

### 2.2 RNA-sequencing

Total RNA was extracted from epWAT of *Ireb2* ^D826V/D826V^ mice and WT mice, each group comprising three male specimens, using the Trizol reagent (15596-018, Invitrogen, United States). For RNA sequencing (RNA-seq) analysis, the integrity and concentration of the RNA samples were assessed with the Agilent 2100 Bioanalyzer (Agilent Technologies, CA, United States). Subsequently, complementary DNA (cDNA) libraries were constructed and sequenced on the Novaseq X platform (Hangzhou Astrocyte Technology, China). Each sample yielded more than 6.11 Gb, with base call quality scores exceeding 98.5% for Q20% and 96.04% for Q30. The raw sequencing reads were subjected to quality filtering using Fastp to produce clean reads, which were then aligned to the mouse reference genome (GRCm38/mm10) using HISAT2, achieving mapping rates between 91.85% and 95.1%. A total of 20,162 expressed genes were identified. Differentially expressed genes (DEGs) were determined using DESeq2, applying a false discovery rate (FDR) threshold of less than 0.05 and an absolute log_2_ fold change criterion greater than 1. Data analysis and visualization, incorporating Principal Component Analysis (PCA), were performed utilizing an online platform (https://magic-plus.novogene.com/). The PCA results indicated that genotype constituted the primary source of variation, with the first two principal components (PC1 accounting for 28.8% and PC2 for 23.2%) collectively explaining 52.0% of the total variance. The RNA-Seq data supporting these findings have been deposited in the CNSA public dataset (https://db.cngb.org/cnsa/) under the accession number CNP0008452.

### 2.3 Metabolomic analysis

An untargeted metabolomic analysis was performed on epWAT derived from *Ireb2* ^D826V/D826V^ and WT mice. The analysis employed a high-performance liquid chromatography (HPLC) system in conjunction with a mass spectrometer (Thermo Fisher Scientific, Germany) and utilized a hydrophilic interaction liquid chromatography (HILIC) column (ZIC-pHILIC, 150 × 4.6 mm, 5 μm particle size; Hichrom Ltd., United Kingdom). Data acquisition was managed using the Xcalibur 2.1.0 software package (Thermo Fisher Scientific, United Kingdom), and the MzMatch software (IDEOM) was employed to convert the signal peaks generated by Xcalibur into numerical data for subsequent processing and analysis. Metabolite identification adhered to the Metabolomics Standards Initiative (MSI) levels. KEGG analysis, PCA, GSEA, and differential metabolites analysis were conducted using an online platform (https://www.bioinformatics.com.cn). The data that support the findings of untargeted metabolomic analysis have been deposited into CNSA public dataset (https://db.cngb.org/cnsa/) with accession number CNP0008452.

### 2.4 Western blot

The protein extracted from epWAT and adipocytes were detected by western blot analysis. 100 mg epWAT was excised from mice or 1 × 10^6^ adipocytes induced by pre-adipocytes from SVFs were cracked using 300 μL RIPA lysis buffer (G2002, Servicebio, China) supplemented with 1 mM phenylmethylsulfonyl fluoride (PMSF) (P0100, Solarbio, China) at 4 °C. Subsequently, the cracked mixtures were centrifuged at 12000 g/min for 15 min, and the protein supernatants were collected and protein concentrations were determined using the BCA Protein Quantification Kit (#E112, Vazyme, China). Proteins were separated via 8% or 10% SDS-PAGE gel electrophoresis, and membranes were subsequently blocked with 5% skim milk (GC310001, Servicebio, China). The membranes were then incubated with primary antibodies (all from Proteintech Company, China) specific to IREB2 (1:1000 diluted by TBST, 29976-1-AP), FTH1 (1:1000 diluted by TBST, 11682-1-AP), ACACA (1:1000 diluted by TBST, 21923-1-AP), ACLY (1:1000 diluted by TBST, 15421-1-AP), FASN (1:1000 diluted by TBST, 10624-2-AP), EGR1 (1:1000 diluted by TBST, 55117-1-AP), FGFR4 (1:1000 diluted by TBST, 11098-1-AP), and β-actin (1:3000 diluted by TBST, 20536-1-AP), followed by incubation with HRP-conjugated goat anti-rabbit (1:3000 dilution, AS014) and HRP-conjugated goat anti-mouse (1:3000 diluted by TBST, AS115) secondary antibodies (Abclonal Company, China). Relative protein expression levels were quantified using ImageJ software (National Institutes of Health, the United States).

### 2.5 Section staining and TG, FFA detection

Histological and biochemical analyses were conducted employing methodologies consistent with those utilized in previous studies (Lv et al., 2019; Lv et al., 2025). In summary, epWAT and SAT collected from WT and *Ireb2* ^D826V/D826V^ mice were preserved using 10% neutral-buffered formalin (G1101, Servicebio, China). The preserved tissues were subsequently embedded in paraffin. Sections of 5 μm thickness were prepared using a tissue processor and stained with H&E solution. Further analysis and imaging were conducted utilizing the CaseViewer software (3D HISTECH, Hungary). The concentrations of TG, TC and free fatty acids (FFA) in serum, epWAT, and adipocytes were quantified using specific assay kits (A110-1-1 for TG detection, and A042-2-1 for FFA detection) procured from Nanjing Jiancheng Company as the instructions.

### 2.6 Iron detection in adipose tissues

Iron levels in adipose tissue were measured using Abcam’s iron assay kits (ab83366) following the instructions. Adipose tissues (0.1 g) were homogenized with 1 mL of iron assay buffer, then centrifuged at 16,000 rpm, 4 °C for 10 min. The supernatant’s absorbance at OD 570 nm was read using 96-well microplate readers. Total iron levels were calculated as per the manufacturer’s guidelines.

### 2.7 Induction of mature adipocytes and iron chelator treatment

According to published reports, pre-adipocytes were isolated from the stromal vascular fractions (SVFs) of *Ireb2* ^D826V/D826V^ and WT mice (Yang et al., 2020; Fang et al., 2024). These cells were cultured in Dulbecco’s Modified Eagle Medium (DMEM) (SH30022.01B, Hyclone, United States) supplemented with 1% antibiotics (G4003, Servicebio, China) and 10% fetal bovine serum (FBS) (E600001, Sangon Biotech, China) in a humidified incubator set at 37 °C with ambient oxygen levels and 5% CO_2_. Differentiation of *Ireb2* ^D826V/D826V^ and WT pre-adipocytes into adipocytes was induced using media containing insulin (HY-P73243, 10 mg/mL, Medchemexpress, China), dexamethasone (HY-14648,10 μM, Medchemexpress, China), 3-isobutyl-1-methylxanthine (HY-12318, IBMX, 0.5 M, Medchemexpress, China), indomethacin (HY-14397, 125 nM, Medchemexpress, China), and rosiglitazone (HY-17386, 1 μM, Medchemexpress, China) for 5 days, followed by treatment with insulin (10 mg/mL) for an additional 2 days. For the treatment with the iron chelator deferoxamine (HY-B1625, Medchemexpress, China) at a concentration of 30 μM, *Ireb2* ^D826V/D826V^ cells were induced using the aforementioned media supplemented with deferoxamine. Post-induction, the adipocytes were subjected to oil red O staining, TG and FA level detection, fluorescence quantitative PCR and Western blot analysis.

### 2.8 Statistical analysis

Student’s t-test was employed to assess the differences between groups. Data are expressed as means ± standard error. Subsequent statistical analyses were performed using Tukey’s post hoc test, utilizing GraphPad Prism 8.0 software. A P-value of less than 0.05 was considered to indicate statistical significance. The sample size (n = 5 per genotype) for this initial phenotypic study was determined based on common practice in exploratory studies of novel mouse models and in adherence to the 3R principle of Reduction. While an *a priori* power analysis was not conducted, a post hoc analysis for the primary outcome (body weight at 16 weeks) revealed that the observed large effect size (Cohen’s *d* = 4.03) resulted in a statistical power >99.9% (α = 0.05), indicating the sample was sufficient to detect the reported phenotype.

## 3 Results

### 3.1 *Ireb2* ^D826V/D826V^ mutation increased the body weight and fat content of mice

**FIGURE 1:**
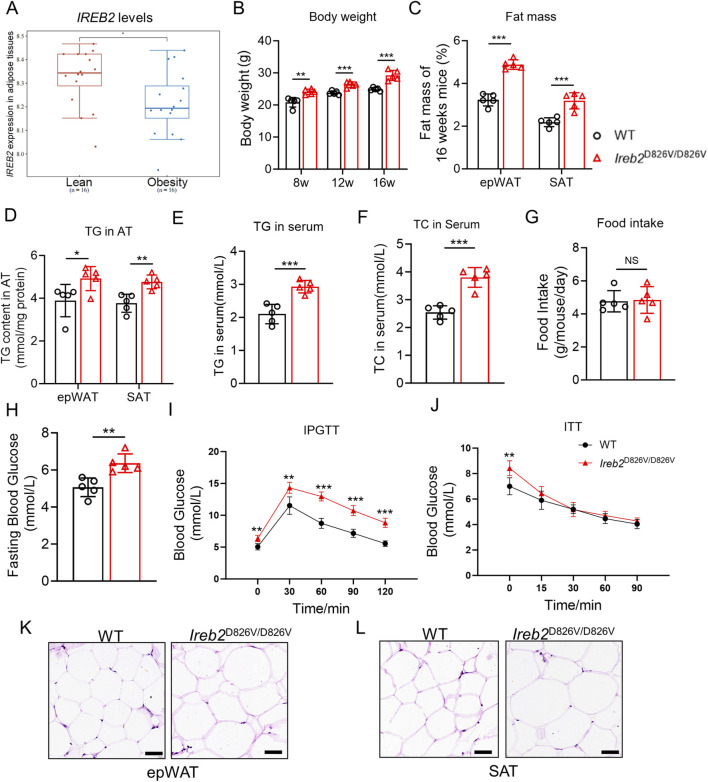
The *Ireb2* ^D826V/D826V^ mutation results in increased body weight and lipid content in mice. **(A)** Human *IREB2* mRNA expression is significantly reduced in obese individuals compared to lean individuals (n = 16 biological replicates for both lean and obese groups, *P < 0.05, relative to the lean group); **(B)** Mice with the *Ireb2* ^D826V/D826V^ mutation exhibit greater body weight compared to WT mice; **(C)** 16 weeks *Ireb2* ^D826V/D826V^ mutant mice demonstrate increased fat mass in epWAT and SAT relative to WT mice; **(D)** Triglyceride (TG) content is elevated in the epWAT and SAT of *Ireb2* ^D826V/D826V^ mutant mice compared to WT mice; **(E)** Serum TG content is also increased in *Ireb2* ^D826V/D826V^ mutant mice relative to WT mice; **(F)** Serum total cholesterol is increased in *Ireb2* ^D826V/D826V^ mutant mice; **(G)** Food intake remains comparable between *Ireb2* ^D826V/D826V^ mutant mice and WT mice; **(H)** *Ireb2* ^D826V/D826V^ mutation slightly increased the fasting blood glucose level; **(I)** IPGTT detection and **(J)** ITT detection for WT and *Ireb2* ^D826V/D826V^ mice; **(K)** Hypertrophic adipocytes are observed in the epWAT of *Ireb2* ^D826V/D826V^ mutant mice compared to WT mice; **(L)** Similarly, hypertrophic adipocytes are present in the SAT of *Ireb2* ^D826V/D826V^ mutant mice compared to WT mice (scale bar = 50 μm, B-J n = 5 biological replicates, *P < 0.05, **P < 0.01, ***P < 0.001, relative to the WT group using Student’s t-test).

To establish the correlation between *IREB2* expression and body weight gain, the human *IREB2* mRNA expression levels in adipose tissues from 16 lean and 16 obese patients were analyzed (GSE59034). As illustrated in Figure 1A, adipose tissues from obese individuals exhibited significantly lower *IREB2* mRNA expression compared to those from lean individuals. This suggests that *IREB2* gene may play a negative regulatory role in the development of obesity. During the feeding period for our established *Ireb2* ^D826V/D826V^ mice, it was observed that *Ireb2* ^D826V/D826V^ mice exhibited increased body weight compared to age-matched WT mice (Figure 1B). Subsequent analysis of fat mass revealed that *Ireb2* ^D826V/D826V^ mice possessed greater amounts of epWAT and SAT compared to WT mice (Figure 1C). Similarly, *Ireb2* ^D826V/D826V^ mice exhibited an increase in epWAT, SAT, and liver weight, while maintaining comparable gastrocnemius muscle weight relative to WT mice (Table 1). This suggests that the *Ireb2* ^D826V/D826V^ mutation may affect the weight of tissues associated with lipid accumulation. Furthermore, the TG concentrations in epWAT (Figure 1D), as well as the TG (Figure 1E) and TC concentrations (Figure 1F) in the serum of *Ireb2* ^D826V/D826V^ mice, exhibited trends similar to those observed in fat mass and body weight. Despite these observations, the *Ireb2* ^D826V/D826V^ mice exhibited food intake amounts comparable to those of WT mice (Figure 1G). The *Ireb2* ^D826V/D826V^ mice exhibited a modest elevation in fasting blood glucose levels (Figure 1H) and impaired glucose tolerance (Figure 1I), while maintaining comparable insulin tolerance to WT mice (Figure 1J). This suggests that the obesity development associated with the *Ireb2* ^D826V/D826V^ mutation is accompanied by dysfunctions in glucose metabolism and a slight degree of insulin resistance. Additionally, hypertrophic adipocytes were identified in the HE-stained sections of epWAT and SAT in *Ireb2* ^D826V/D826V^ mice, as compared to WT mice (Figures 1K,L). These findings imply that the *Ireb2* ^D826V/D826V^ mutation may facilitate lipid accumulation without an increase in energy intake, but the potential mechanism remains obscure.

**TABLE 1:** Body weights and tissues weights of mice, n = 5, ***P < 0.001, compared to the WT group using Student’s t-test.

Tissues	WT	*Ireb2* ^D826V/D826V^	P Value
epWAT	0.80 ± 0.07 g	1.43 ± 0.10g	<0.001
SAT	0.54 ± 0.06 g	0.93 ± 0.10g	<0.001
Liver	1.27 ± 0.06 g	1.52 ± 0.05g	<0.001
Gastrocnemius muscle	0.70 ± 0.03 g	0.68 ± 0.06g	0.51
Body weight	24.88 ± 0.51 g	29.22 ± 1.4g	<0.001

### 3.2 *Ireb2* ^D826V/D826V^ mutation activated the lipid biosynthetic process in epWAT

**FIGURE 2:**
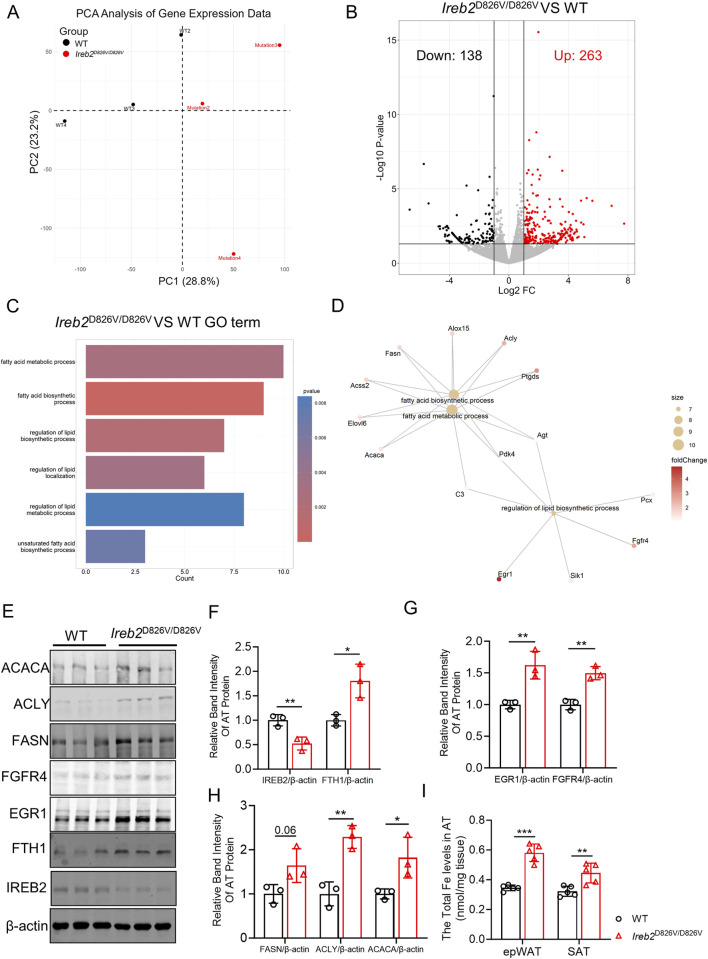
The *Ireb2* ^D826V/D826V^ mutation led to an increase in the expression of lipid-related genes in epWAT. **(A)** Principal Component Analysis (PCA) was conducted on the epWAT of WT and *Ireb2* ^D826V/D826V^ mutant mice using RNA sequencing data; **(B)** A volcano plot illustrated that the *Ireb2* ^D826V/D826V^ mutation resulted in the upregulation of 263 genes and the downregulation of 138 genes compared to WT mice; **(C)** Gene Ontology (GO) term analysis of the 263 upregulated genes indicated an enrichment of functions related to lipid biosynthesis; **(D)** There is a correlation between the terms “fatty acid biosynthesis” and “regulation of lipid biosynthetic process”; **(E)** Western blot analysis was performed to assess the expression of proteins related to fatty acid biosynthesis, triglyceride biosynthesis, IREB2, and FTH1; **(F)** Relative band intensity analysis was conducted for IREB2 and FTH1 proteins; **(G)** Relative band intensity analysis was performed for EGR1 and FGFR4 proteins; **(H)** Relative band intensity analysis was carried out for FASN, ACACA, and ACLY proteins; **(I)** Fe levels in epWAT and SAT of WT and *Ireb2* ^D826V/D826V^ mutant mice (A-H n = 3 biological replicates, I n = 5 biological replicates, *P < 0.05, **P < 0.01, ***P < 0.001, compared to the WT group using Student’s t-test).

To elucidate the potential mechanism by which the *Ireb2* ^D826V/D826V^ mutation influences lipid accumulation, epWAT from both *Ireb2* ^D826V/D826V^ and WT mice was collected and subjected to RNA sequencing analysis. The resulting RNA sequencing data were subsequently examined using principal component analysis (PCA) (Figure 2A), and samples of WT and *Ireb2* ^D826V/D826V^ group showed qualified separation. Differential gene expression analysis revealed that, in comparison to WT mice, 263 genes were upregulated and 138 genes were downregulated in the epWAT of *Ireb2* ^D826V/D826V^ mice (Figure 2B). To further elucidate the functional implications of these differentially expressed genes (DEGs), the 263 upregulated DEGs were analyzed using the Gene Ontology (GO) method. As illustrated in Figures 2C,D, these upregulated DEGs were significantly enriched in functions related to lipid biosynthesis, including the fatty acid biosynthetic process and the regulation of lipid biosynthetic processes, among others. Based on the DEGs associated with fatty acid and lipid biosynthesis identified through RNA sequencing, the functional characterization of these genes was assessed via western blot analysis. As illustrated in Figures 2E–H, there was an upregulation of fatty acid-related enzymes, such as ACACA, ACLY, and FASN proetins, as well as triglyceride-related proteins, including FGFR4 and EGR1, in the epWAT of *Ireb2* ^D826V/D826V^ mice. This suggests that the *Ireb2* ^D826V/D826V^ mutation enhances lipid biosynthetic function by upregulating enzymes associated with lipid biosynthesis. Building on our previous study, which identified the D826V mutation as a trigger for IREB2 protein degradation (Guo et al., 2024), we conducted an analysis of IREB2 protein expression levels in epWAT. Our findings revealed a marked decrease in IREB2 protein levels and an increase in FTH1 protein levels compared to WT mice, which was identified with the previous report (Yang et al., 2025). The upregulation of FTH1, a critical protein involved in iron metabolism, led to an increase in intracellular iron levels, which was associated with decreased energy expenditure and augmented lipid biosynthesis (Lu et al., 2024; Wang et al., 2025). Our findings indicated that iron levels were elevated in the epWAT and SAT of *Ireb2* ^D826V/D826V^ mice compared to WT mice, which corresponded with decreased IREB2 protein levels and increased FTH1 expression (Figure 2I). Based on these findings, we hypothesize that the disruption of iron metabolism in epWAT caused by the *Ireb2* ^D826V/D826V^ mutation enhances the biosynthetic processes of fatty acids and triglycerides.

### 3.3 Metabolomics identified that *Ireb2* ^D826V/D826V^ mutation increased the lipid biosynthetic process

**FIGURE 3:**
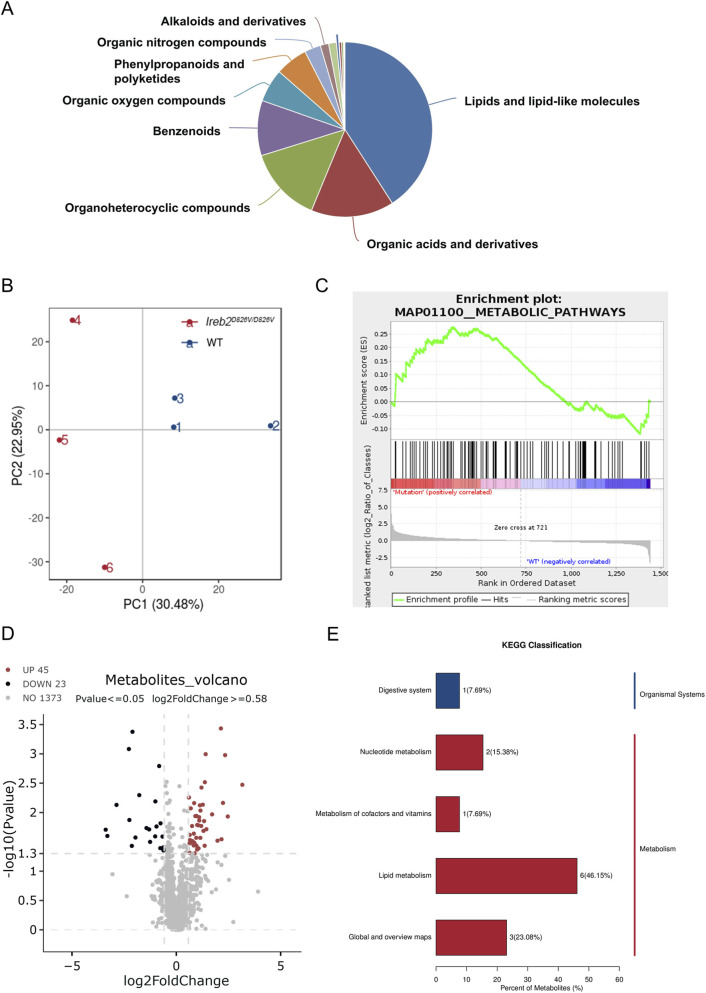
Metabolomic Analysis of epWAT in *Ireb2* ^D826V/D826V^ Mutant and WT Mice. **(A)** Lipids and lipid-like molecules constituted a significant proportion of the metabolites identified in epWAT. **(B)** PCA was conducted on the metabolomic profiles of epWAT from both WT and *Ireb2* ^D826V/D826V^ mutant mice. **(C)** Gene Set Enrichment Analysis (GSEA) revealed that metabolic pathways were positively correlated with the *Ireb2* ^D826V/D826V^ mutation. **(D)** A volcano plot illustrated that the *Ireb2* ^D826V/D826V^ mutation resulted in the upregulation of 45 metabolites and the downregulation of 23 metabolites compared to WT mice. **(E)** Kyoto Encyclopedia of Genes and Genomes (KEGG) classification indicated that 46.15% of the metabolites were associated with the lipid metabolism category; (A-E n = 3 biological replicates).

**FIGURE 4:**
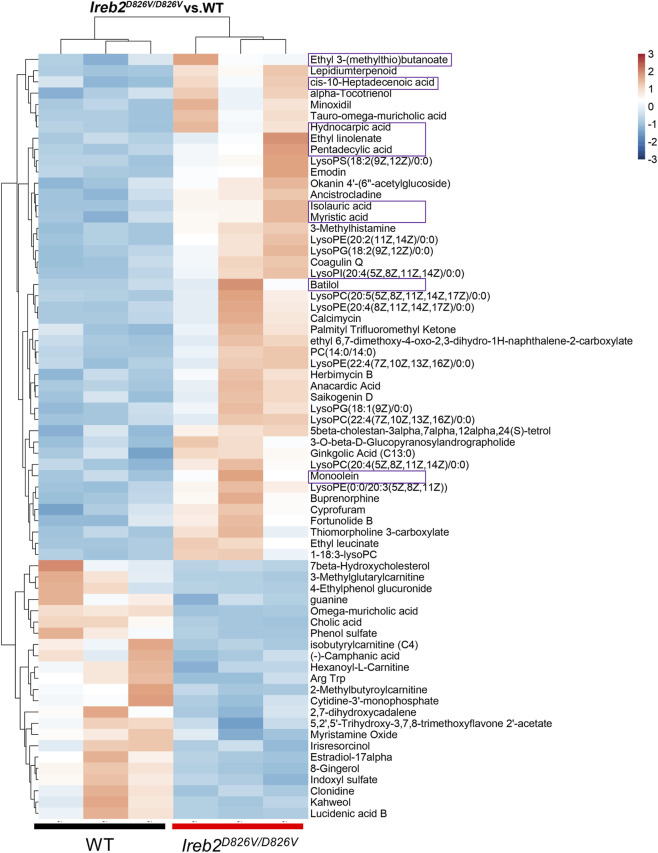
The heatmap illustrates the differential metabolites in epWAT of *Ireb2* ^D826V/D826V^ mutant and WT mice. Purple rectangles indicate the upregulated fatty acids and components involved in triglyceride biosynthesis in *Ireb2* ^D826V/D826V^ mutant mice; n = 3 biological replicates.

To determine whether the increased expression of lipid biosynthetic enzymes influenced the metabolic profile in epWAT, metabolomic analyses were performed using epWAT samples from *Ireb2* ^D826V/D826V^ and WT mice. As illustrated in Figure 3A, lipids and lipid-like molecules constituted a significant portion of the total metabolites. Principal component analysis (PCA) of the metabolites revealed distinct metabolic profiles between *Ireb2* ^D826V/D826V^ and WT mice (Figure 3B). Furthermore, gene set enrichment analysis (GSEA) of the metabolomics data indicated a positive association between the *Ireb2* ^D826V/D826V^ mutation and metabolic pathways (Figure 3C). Subsequent differential metabolites analysis identified 45 upregulated and 23 downregulated metabolites (Figure 3D). KEGG pathway enrichment analysis further classified the majority of these metabolites as related to lipid metabolism (Figure 3E). To determine whether the *Ireb2* ^D826V/D826V^ mutation leads to an increase in fatty acid and triglyceride biosynthesis, a heatmap was used to illustrate various metabolites (Figure 4). Fatty acids such as cis-10-Heptadecenoic acid, hydnocarpic acid, pentadecylic acid, isolauric acid, and myristic acid were significantly elevated in the epWAT of *Ireb2* ^D826V/D826V^ mice. Additionally, other lipid biosynthesis components, including Ethyl 3-(methylthio)butanoate, ethyl linolenate, batilol, and monoolein, also showed increased levels in these mice. These findings suggest that the *Ireb2* ^D826V/D826V^ mutation enhances lipid biosynthesis in epWAT, potentially due to the upregulation of lipid-related enzymes.

### 3.4 *Ireb2* ^D826V/D826V^ mutation increased the lipid biosynthesis *in vitro*

**FIGURE 5:**
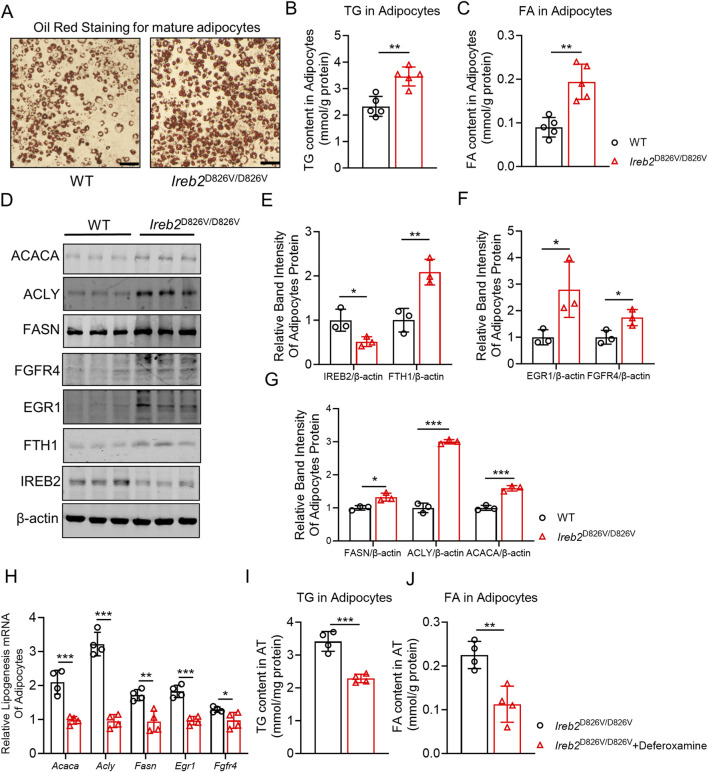
*In vitro* experiments demonstrated that the *Ireb2* ^D826V/D826V^ mutation enhances lipid biosynthesis in mature adipocytes. **(A)** Oil Red O staining was performed on WT and *Ireb2* ^D826V/D826V^ mature adipocytes; **(B)** The *Ireb2* ^D826V/D826V^ mutation led to an increase in TG content in mature adipocytes; **(C)** The mutation also elevated fatty acid content in mature adipocytes; **(D)** Western blot analysis was conducted to assess the expression of proteins related to fatty acid and triglyceride biosynthesis, as well as IREB2 and FTH1, in mature adipocytes; **(E)** Relative band intensity analysis was carried out for IREB2 and FTH1 proteins; **(F)** Relative band intensity analysis was conducted for EGR1 and FGFR4 proteins; **(G)** Relative band intensity analysis was performed for FASN, ACACA, and ACLY proteins; **(H)** Lipogenic related mRNA expression in *Ireb2* ^D826V/D826V^ adipocytes and *Ireb2* ^D826V/D826V^ + deferoxamine adipocytes; **(I)** TG content in *Ireb2* ^D826V/D826V^ adipocytes and *Ireb2* ^D826V/D826V^ + deferoxamine adipocytes; **(J)** FA content in *Ireb2* ^D826V/D826V^ adipocytes and *Ireb2* ^D826V/D826V^ + deferoxamine adipocytes. (A-C n = 5 biological replicates; D-G n = 3 biological replicates, H-J n = 4 biological replicates, *P < 0.05, **P < 0.01, ***P < 0.001, **(A-G)** compared with the WT group using Student’s t-test; **(H-J)** compared with the *Ireb2* ^D826V/D826V^ group using Student’s t-test.

Mature adipocytes derived from primary fibroblasts were utilized to further assess lipogenic capabilities. As depicted in Figure 5A, both *Ireb2* ^D826V/D826V^ and WT adipocytes exhibited mature lipid vacuoles, as evidenced by oil red staining, with *Ireb2* ^D826V/D826V^ adipocytes displaying a greater abundance of lipid vacuoles. Subsequent analyses of TG and FA revealed that *Ireb2* ^D826V/D826V^ adipocytes contained higher levels of TG and FA (Figures 5B,C). Western blot analysis of adipocytes *in vitro* further demonstrated that the mutation *Ireb2* ^D826V/D826V^ led to an upregulation of FA and lipid-associated enzymes (Figures 5D,F,G). Additionally, iron-regulated proteins, including FTH1 and IREB2, exhibited similar trends to those observed in epWAT (Figures 5D,E). To determine whether iron accumulation in adipocytes stimulates the lipogenic process, deferoxamine, a well-established iron chelator, was employed during the differentiation of *Ireb2* ^D826V/D826V^ adipocytes. The application of deferoxamine resulted in the suppression of lipogenic-related mRNA expression in *Ireb2* ^D826V/D826V^ adipocytes (Figure 5H), and both TG (Figure 5I) and FA (Figure 5J) contents were reduced following deferoxamine treatment. This suggests a positive correlation between iron accumulation and the expression of lipogenic mRNAs, including *Acaca*, *Fasn*, *Acly*, *Egr1*, and *Fgfr4*. These findings suggest that the degradation of IREB2, coupled with the upregulated expression of FTH1, is associated with enhanced lipid biosynthesis in adipocytes, potentially as a result of increased iron accumulation.

## 4 Discussion

IREB2, a crucial regulatory protein involved in iron metabolism, specifically binds to the iron-responsive element (IRE), subsequently downregulating the expression of ferritin protein. Inhibition of IREB2 expression leads to an upregulation of FTH1 (the heavy chains of ferritin) expression, thereby augmenting iron storage within cells and reducing the levels of free iron (Yanatori et al., 2021; Yao et al., 2024). Previous study has primarily concentrated on the observation that mutations in *IREB2* lead to the degradation of IREB2 protein (Guo et al., 2024). This reduction subsequently results in enhanced FTH1 protein expression, ferric iron accumulation and mitochondrial dysfunction in neuronal cells, ultimately contributing to the onset of neurodegenerative diseases (Maio et al., 2022; LaVaute et al., 2001). In contrast, this report is the first to elucidate the relationship among IREB2 degradation, FTH1 upregulation, iron accumulation, and the lipid biosynthetic process. This association may be attributed to variations in iron levels influencing the synthesis of fatty acids and triglyceride-associated proteins.

The relationship between *Ireb2* ^D826V/D826V^ mutation and lipid biosynthesis in mice presents an intriguing research question. Currently, there is a lack of published studies elucidating the lipid metabolic functions regulated by the IREB2 protein. It has been observed that inhibition of IREB2 protein expression leads to an increase in ferritin, a crucial protein involved in iron storage. Previous studies have demonstrated that diets rich in iron or increased iron storage are associated with elevated lipid levels, and enhanced FTH1 has been proven positively associated with the obesity development (Wang et al., 2025; Liang et al., 2024; Chen et al., 2024). However, the specific regulatory role of FTH1 and the impact of iron accumulation on the development of obesity remain insufficiently understood. In our study, we initially observed that *Ireb2* ^D826V/D826V^ mice exhibited increased body weights, enlarged adipocyte areas, and elevated iron concentrations in epWAT and SAT, as well as elevated levels of fatty acids and triglycerides in both serum and epWAT. These findings suggest that the *Ireb2* ^D826V/D826V^ mutation induces increased body weight and lipid accumulation, potentially implicating the role of the IREB2 protein in iron accumulation. In conjunction with elevated iron concentrations in epWAT and SAT, our western blot analysis revealed that the *Ireb2* ^D826V/D826V^ mutation promotes the degradation of IREB2 and upregulates FTH1 expression. It is plausible to hypothesize that the *Ireb2* ^D826V/D826V^ mutation leads to upregulated FTH1 expression, which, in conjunction with increased iron accumulation, may contribute to augmented lipid biosynthesis. Previous studies have demonstrated that iron accumulation, regulated by FTH1 expression, contributes to increased lipid accumulation. This process is facilitated by the activation of the sgk-1/FATP1/4 signaling pathway, which enhances the transport of fatty acids into cells (Wang et al., 2016). Nonetheless, concentrating exclusively on fatty acid transport fails to comprehensively capture the lipid accumulation process associated with FTH1 expression and iron accumulation. Therefore, further investigation into the underlying mechanisms is necessary.

The results presented above suggest that the *Ireb2* ^D826V/D826V^ mutation may influence body weight gain and lipid accumulation. Furthermore, the degradation of IREB2 and the upregulation of FTH1 expression, leading to iron accumulation, seem to contribute to these phenomena. Although some published studies have identified this association, the underlying mechanism remains unclear (Hilton et al., 2023; Wang et al., 2016; Festa et al., 2000). To explore the potential mechanisms by which the *Ireb2* ^D826V/D826V^ mutation affects lipid metabolism, RNA sequencing was performed. The findings indicated a significant increase in fatty acid or lipid biosynthesis process in *Ireb2* ^D826V/D826V^ mice, indicating *Ireb2* ^D826V/D826V^ mutation increased the lipid accumulation by enhancing fatty acid or lipid biosynthesis in epWAT. The findings initially indicated that the *Ireb2* ^D826V/D826V^ mutation specifically activated enhanced lipid biosynthesis, rather than other pathways, and led to increased FTH1 expression and iron accumulation. However, the specific protein involved in regulating these processes remains unidentified. Previous research has demonstrated that the upregulation of FTH1 expression and subsequent iron accumulation, induced by the downregulation of IREB2 protein, is positively associated with elevated lipid levels and the onset of obesity (Hilton et al., 2023; Wang et al., 2016; Kim et al., 2022). This relationship may be partially attributed to ferritin’s ability to bind fatty acids, thereby protecting them from oxidation (Bu et al., 2012). Furthermore, elevated ferritin expression reduces free iron ion levels and inhibits ferroptosis by curtailing the lipid peroxidation process. This undoubtedly creates an environment conducive to lipid accumulation (Xiong et al., 2025). However, the specific lipid-related molecular mechanisms regulated by FTH1 remain unclear. In this study, we observed an upregulation of the fatty-acid biosynthetic proteins ACACA, ACLY, and FASN, as well as the triglyceride biosynthetic proteins EGR1 and FGFR4, in the epWAT of *Ireb2* ^D826V/D826V^ mice and their mature adipocytes, which identified by RNA-sequencing and western blot. These fatty acid and triglyceride biosynthetic proteins upregulation occurred alongside decreased IREB2 expression and increased FTH1 expression, supposing that the *Ireb2* ^D826V/D826V^ mutation may enhances the expression of these lipid biosynthesis proteins. In the context of fatty acid biosynthesis, ACACA, ACLY, and FASN are significant enzymes (Chen et al., 2023). ACACA (Acetyl-CoA Carboxylase) catalyzes the initial and rate-limiting step by converting acetyl-CoA into malonyl-CoA, which is a crucial precursor for fatty acid synthesis (Harriman et al., 2016). ACLY (ATP Citrate Lyase) provides the fundamental substrate by cleaving citrate derived from mitochondria into cytosolic acetyl-CoA, thereby linking carbohydrate metabolism to lipid production (Mukhi et al., 2023). FASN (Fatty Acid Synthase) functions as the central enzyme complex responsible for synthesizing palmitate through the sequential addition of two-carbon units from malonyl-CoA to an elongating fatty acid chain (Xu et al., 2024). The synchronized upregulation of these three critical enzymes facilitates the process of fatty acid synthesis. This enhanced expression seems to be initiated by iron accumulation resulting from the *Ireb2* ^D826V/D826V^ mutation and the consequent increase in FTH1 levels, although the exact mechanism has not yet been elucidated in the literature. Our study demonstrated that the administration of an iron chelator to *Ireb2* ^D826V/D826V^ mutant adipocytes led to a reduction in the mRNA expression of all three enzymes to varying extents. This finding suggests that their expression levels are modulated by elevated intracellular iron concentrations. Earlier studies indicate that increased iron levels can enhance the expression of transcription factors like PPARγ, which are responsible for regulating these three enzymes and the fatty acid biosynthetic process (Song et al., 2022). Thus, it is proposed that these transcription factors might play a role in controlling the three main enzymes involved in fatty acid synthesis, a link that should be explored in future research.

The *Ireb2* ^D826V/D826V^ mutation also leads to an upregulation of triglyceride biosynthetic proteins, such as EGR1 and FGFR4. EGR1, a transcription factor, is crucial in modulating the expression of various genes involved in lipid storage (Zhang et al., 2013; Chakrabarti et al., 2013). Notably, EGR1 directly suppresses the transcription of adipose triglyceride lipase (ATGL) in adipocytes, thereby reducing lipolysis and promoting triglyceride accumulation (Meriin et al., 2022). This process ultimately contributes to excessive lipid deposition within adipose tissue. Furthermore, the accumulation of iron demonstrated a positive correlation with EGR1 expression (Xu et al., 2025), and *Erg1* mRNA decreased in the *Ireb2* ^D826V/D826V^ mutated adipocytes after deferoxamine treatment, suggesting a compelling hypothesis: iron accumulation, modulated by the upregulation of FTH1 expression in *Ireb2* ^D826V/D826V^ mice, may play a role in the increased expression of EGR1. Conversely, FGFR4 is recognized for its direct involvement in adipocyte development and triglyceride synthesis, with FGFR4 knockdown significantly reducing triglyceride levels in adipocytes (Huang et al., 2020). Currently, the literature lacks direct evidence elucidating the impact of iron accumulation on FGFR4 expression, highlighting an area that warrants further investigation.

Moreover, the upregulation of lipid-biosynthetic proteins was substantiated by metabolomic analyses, which revealed an increase in specific fatty acids and precursors involved in triglyceride biosynthesis within the epWAT of *Ireb2* ^D826V/D826V^ mice. This finding was further supported by the elevated levels of triglycerides and fatty acids observed in mature adipocytes. Collectively, these results not only affirm enhanced lipid production but also establish a direct link between the altered molecular landscape and the expansion of adipose tissue, as well as the systemic obesogenic phenotype observed in *Ireb2* ^D826V/D826V^ mice.

## 5 Conclusion

In conclusion, this study identifies the *Ireb2* ^D826V/D826V^ mutation as a novel genetic determinant influencing obesity and lipid metabolic reprogramming. We elucidate a pathogenic sequence wherein the mutation induces destabilization of the IREB2 protein, leading to the upregulation of FTH1 and potential cellular iron accumulation. This iron-rich state is associated with the concomitant upregulation of key genes involved in fatty acid synthesis (ACACA, ACLY, FASN) and triglyceride accumulation (EGR1, FGFR4). This coordinated gene expression pattern is correlated with increased lipid biosynthesis and weight gain, suggesting a potential molecular link between iron storage and metabolic phenotype. These findings associate the IREB2-FTH1-Iron Accumulation axis with systemic metabolic alterations in energy homeostasis and adiposity, suggesting a link that warrants future mechanistic investigation.

## 6 Limitations and future perspectives

This study acknowledges several limitations that suggest significant avenues for future research. Firstly, although we propose a connection between IREB2 degradation, FTH1-mediated iron accumulation, and the upregulation of key lipogenic enzymes, the precise mechanistic pathway through which increased cellular iron levels transcriptionally regulate lipid synthesis necessitates further experimental validation. Secondly, to definitively elucidate the regulatory relationship between iron metabolism and lipid metabolism as established in this study, future research should employ IREB2-deficient and FTH1-deficient mouse models to enable more direct genetic manipulation.

## Data Availability

The data presented in the study are deposited in the CNSA public data set (https://db.cngb.org/cnsa/), accession number CNP0008452.
